# The Potential of Sound Analysis to Reveal Hemodynamic Conditions of Arteriovenous Fistulae for Hemodialysis

**DOI:** 10.1007/s10439-024-03638-2

**Published:** 2024-11-01

**Authors:** Sofia Poloni, Luca Soliveri, Anna Caroli, Andrea Remuzzi, Michela Bozzetto

**Affiliations:** 1 Department of Engineering and Applied Sciences, University of Bergamo, Dalmine, Italy; 2 Department of Biomedical Engineering, Istituto di Ricerche Farmacologiche Mario Negri IRCCS, Bergamo, Italy; 3 Department of Management, Information and Production Engineering, University of Bergamo, Dalmine, Italy

**Keywords:** Arteriovenous fistula, Blood flow volume, Hemodialysis, Hemodynamic conditions, Sound analysis

## Abstract

**Purpose:**

Arteriovenous fistula (AVF), the preferred vascular access for hemodialysis, is associated with high failure rate. The aim of this study was to investigate the potential of AVF sound auscultation in providing quantitative information on AVF hemodynamic conditions.

**Methods:**

This single-center prospective study involved six patients with native radio-cephalic AVFs who underwent multiple follow-up visits. Doppler Ultrasound blood flow volume (BFV) assessment and electronic stethoscope-based sound recordings were performed during each visit, whereas MRIs were acquired 3 days, 3 weeks and 1 year after surgery. Computational fluid dynamic (CFD) simulations were performed on patient-specific MRI-derived geometrical models.

**Results:**

Higher values of median peak amplitudes ratios (high-low peak ratio-HLPR) were found to be associated with complex blood flow and velocity streamlines recirculation at systolic peak, and corresponding extended regions of high oscillatory shear index (OSI). On the contrary, lower values of HLPR were associated with laminar flow pattern and low values of OSI. Significant differences were observed in HLPR between subgroups with extended or limited areas with OSI > 0.1 (0.67 vs 0.31, respectively). Significant relationships were found between AVF sound intensity and brachial BFV (slope = 0.103, *p* < 0.01) as well as between longitudinal changes in brachial BFV and HLPR (slope = − 0.001, *p* < 0.01).

**Conclusion:**

Our results show that AVF sound can be exploited to extract fundamental information on AVF hemodynamic conditions, providing indication of the presence of complex hemodynamic and adequate BFV to perform hemodialysis. Sound analysis has therefore the potential to improve clinical AVF surveillance and to ameliorate outcome.

**Supplementary Information:**

The online version contains supplementary material available at 10.1007/s10439-024-03638-2.

## Introduction

End-stage renal disease (ESRD) is a growing global health concern that is closely related to an aging population and longer survival of patients who are reliant on renal replacement therapy. Hemodialysis treatment is necessary for ESRD patients’ survival [1]. Successful hemodialysis requires functional vascular access to provide safe and long-lasting connection between the patient’s circulatory system and the artificial kidney [2]. However, vascular access dysfunction remains the primary contributor to morbidity and hospitalization among patients undergoing hemodialysis, and the major limitation to the treatment’s effectiveness [3]. The current recommendation for vascular access is the native arteriovenous fistula (AVF), which is surgically created in the arm through anastomosis between a vein and an artery [4]. Despite being the first-choice treatment [5], the AVF is still associated with high non-maturation and early failure rates [6] requiring the creation of a new vascular access in most cases [7].

The prevailing hypothesis is that increased hemodynamic stresses resulting from AVF creation can promote a vascular remodeling process called AVF maturation, which consists of an increase in wall thickness and a widening of the vessel’s diameter to restore physiological stress conditions. However, not all AVFs undergo a successful maturation process. Furthermore, in those AVFs that mature successfully, stenotic inward remodeling may occur at a later stage, resulting in vessel lumen occlusion and subsequent AVF failure. The formation of stenosis in the juxta-anastomotic vein is due to neointimal hyperplasia, a complex proinflammatory response that triggers the activation, proliferation, and migration of smooth muscle cells within the intima layer [8, 9]. Although the exact mechanism underlying stenosis development remains unclear [10], there is a general consensus that hemodynamic conditions play a key role in the vascular remodeling process [11].

Over the past few decades, medical image-based computational fluid dynamics (CFD) has been used to quantify local stresses and investigate the role of hemodynamics in AVF remodeling and failure processes. Previous CFD investigations performed by our group on patient-specific AVF geometrical models revealed transitional turbulent-like flow in the vein [12, 13]. More specifically, we identified values of the oscillatory shear index (OSI), a widely recognized hemodynamic measure for identifying disrupted flow conditions, that exceeded 0.1 in significant portions of the venous segment [13]. Moreover, longitudinal studies from our group and others have tried to correlate hemodynamics and vascular remodeling [14, 15], but no definitive correlation between any specific WSS metric and AVF failure has been conclusively established so far [16]. Therefore, although it is clear that the non-physiological transitional flow within the vein is an important factor, further investigation is needed to shed more light on the mechanisms underlying AVF remodeling and failure.

Currently, the incomplete understanding of the physiopathological mechanisms is accompanied by the lack of valuable and effective strategies to monitor AVF function and timely identify AVF stenosis onset. Indeed, AVF monitoring still relies on the measurement of AVF blood flow rate using Doppler Ultrasound (US). However, its widespread application for continuous monitoring of all patients in hemodialysis centers is hindered by time constraints and limited availability, specifically in satellite dialysis centers. During routine clinical practice, the well-functioning of the AVF is more often assessed through physical examination, which include palpation of AVF vessels and auscultation of the sounds [17]. AVF sound auscultation through a stethoscope can be an excellent non-invasive and inexpensive surveillance method [18]. However, so far, sound evaluation remains qualitative and therefore subjective. Recent works [19–21] have suggested that distinctive characteristics of the sound produced by AVF is associated with stenosis. Specifically high-frequency sound up to 700–800 Hz occurs when the bloodstream is subjected to resistance due to vessel stenosis onset [19]. However, the potential of AVF sound has yet to be demonstrated. Therefore, the aim of the current study was to investigate the potential of AVF sound analysis in providing quantitative information on AVF hemodynamic conditions.

## Materials and Methods

### Study Population and Design

This single-center prospective study, conducted at the Nephrology and Dialysis Unit of the Papa Giovanni XXIII Hospital (Bergamo, Italy) included six patients aged between 18 and 75 who were referred for native radio-cephalic AVFs. The study protocol (NCT04141852) received approval from the Local Ethics Committee (Reg 107/19), and all participants provided written informed consent. Exclusion criteria were contraindication for magnetic resonance imaging (MRI) examination, ineligibility for autogenous AVF creation, or presence of a prior ipsilateral vascular access.

**Fig. 1:**
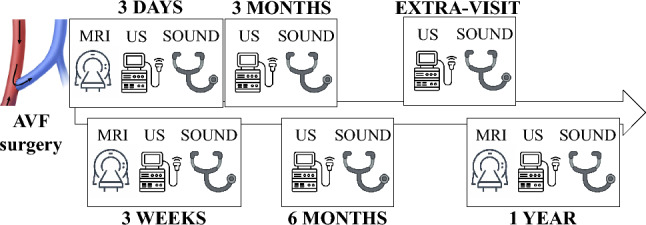
Flow diagram of the study. *AVF* arteriovenous fistula, *MRI* magnetic resonance imaging, *US* ultrasound

Patients underwent follow-up visits at 3 days, 3 weeks, 3 months, 6 months and 1 year after surgery, with the possibility of an additional visit during the ninth month, as shown in Fig. 1. Doppler US and AVF sound recordings were performed during each examination at the Unit of Nephrology and Dialysis, whereas MRI scans were acquired at 3 days, 3 weeks and 1 year after AVF surgery in the Radiology Unit in the Papa Giovanni XXIII Hospital. Supplementary Table 1 details the examinations that each patient underwent during individual visits. Specifically, patients P1, P2 and P4 underwent all examinations and attended all scheduled appointments, and P2 and P4 also attended the 9-month appointment. Patient P3 missed the 3-month follow-up visit due to the COVID-19 lockdown, and for the same reason, patient P6 missed the 3-week follow-up. Finally, patients P5 and P6 were excluded from the hemodynamic analysis due to inadequate AVF image quality and incomplete MRI acquisition due to claustrophobia, respectively. Therefore, comparative analyses involving US measurements and acoustic data were conducted for all patients, whereas CFD analyses and related analysis of hemodynamics and AVF sounds were performed only in patients with complete and high-quality MRI scans (P1-P4).

### AVF Sound Recording and Analysis

Sound recordings of AVFs were acquired with a 3M Littmann 3200 Electronic Stethoscope [22] at the anastomosis site with recordings longer than 5 s. In a subgroup of measurements, AVF sounds were recorded by both an expert and three inexperienced operators to investigate how much sound recording depends on operator expertise. A significant correlation was found between the HLPRs of AVF sounds recorded by the experienced and inexperience operators (R = 0.96, *p* < 0.01), as shown in Supplementary Fig. 1. The Bland–Altman plot also confirmed the consistency between the measures, except for a single outlier.

In all the measurements, special care was taken to maintain the same position for acoustic recordings for each patient throughout the study. Efforts were made to eliminate environmental noise by acquiring the recordings in quiet environment with the door closed to avoid voices and movements that could overwhelm the tones of interest. Any friction between the device and the patient’s skin that could mask the distinctive tones of the sounds under investigation was prevented. Additionally, precautions were taken to avoid applying excessive pressure to the patient’s skin during recording by maintaining a light touch, as the microphone is very sensitive to changes in pressure, as are all pressure transducers. This prevented alterations in hemodynamics and ensured the sounds under investigation were not masked or distorted.

**Fig. 2:**
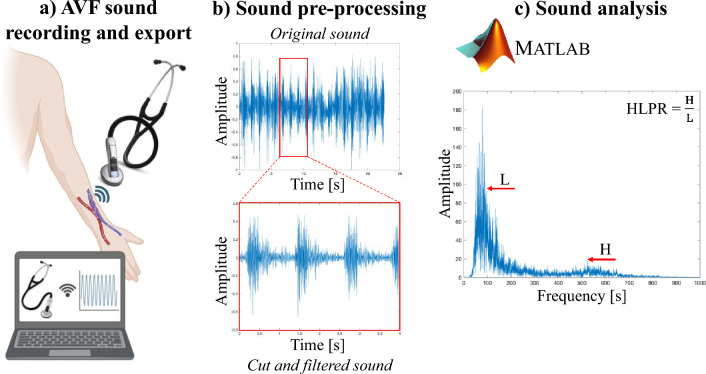
AVF sound recording and analysis procedure. **a** AVF, sound recording and export; **b** Audio tracks pre-processing; **c** Sound analysis in MATLAB. *HLPR* high-low peak ratio, *H* high frequency peak amplitude, *L* low frequency peak amplitude

The audio tracks were subsequently transferred to a computer using the Bluetooth connection between the electronic stethoscope and its App. When saving the audio, the stethoscope’s filter amplified sounds within the range of 20 to 2000 Hz, and reduced frequency responses between 50 and 500 Hz. The AVF sound recording, and export procedure is illustrated in Fig. 2a.

Then, the AVF sounds were pre-processed and analyzed using MATLAB, software version R2022a. Pre-processing consisted of cutting the audio tracks by obtaining a standard duration of 4 s for each recording and filtering with a fourth order high-pass Butterworth filter at 50 Hz to delete the noise introduced by the device (Fig. 2b). The filter was chosen for its flat frequency response in the passband and the steeper transition band.

The spectral features of each AVF sound were analyzed and the frequency domain was obtained by using the Fast Fourier Transform. The frequency and amplitude of the maximum peak in the low (100–250 Hz) and high (500–700 Hz) frequency intervals were identified. The high-low peak ratio (HLPR), a newly proposed metric designed to account high-frequency components in sounds, was calculated as the ratio between the amplitude of maximum peak in the high and low frequency ranges for each sound. AVF sound analysis is shown in Fig. 2c.

### Doppler Ultrasound Acquisition

An experienced nephrologist performed US imaging examinations using a linear probe (MyLab Twice, Esaote). The evaluation of arterial diameters and time-averaged velocity included the brachial artery (BA) in the middle-arm and the radial artery in both proximal (PA) and distal (DA) positions relative to the anastomosis. Diameter measurements were obtained by examining B-mode images of the vessels in their transverse orientation, including measurements of both short and long axes. These measures were subsequently used to calculate the equivalent diameter and the cross-sectional area of each vessel. To measure the time-averaged velocity the operator was required to trace three complete cardiac cycles on the Velocity/Time curve derived from pulsed-wave Doppler, and this process was repeated three times. The mean time-averaged velocity multiplied by the cross-sectional area of each vessel was used to calculate blood flow volume (BFV) in each segment. Since the flow in all AVFs was retrograde, with both PA and DA function as afferent vessels, the venous BFV was calculated by adding their values to each other.

### Computational Fluid Dynamic Simulation

**Fig. 3:**
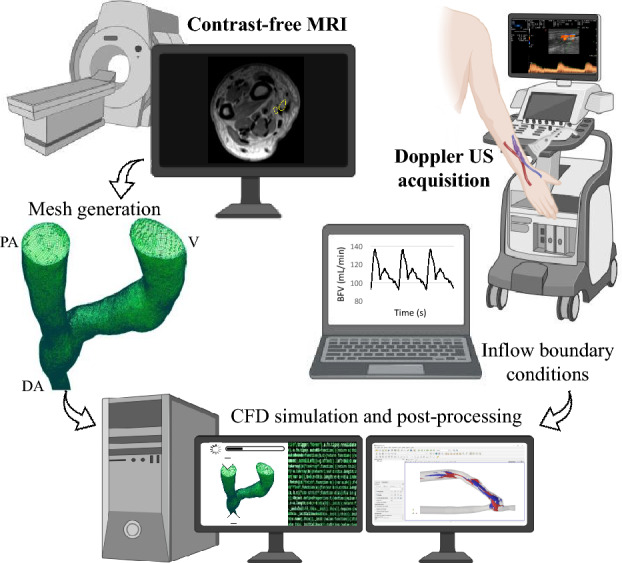
CFD simulation pipeline, from MRI and Doppler US acquisitions to mesh generation, simulation and post-processing. *BFV* blood flow volume, *CFD* computational fluid dynamic, *DA* distal artery, *MRI* magnetic resonance imaging, *PA* proximal artery, *US* ultrasound, *V* vein

The pipeline of the computational study is reported in Fig. 3. All details regarding MRI scan acquisition and CFD simulation methodology have been reported in our earlier publication [23]. Briefly, contrast-free 3D fast spin echo T1-weighted imaging with variable flip angles (CUBE sequence using a 1.5 T scanner GE, Optima 450w GEM) was acquired to obtain high-quality images suitable for reliable vessel segmentation. The patient-specific 3D surface models of the AVFs were generated using the open-source software Vascular Modeling Toolkit (VMTK) [24]. To enhance the surface quality, a smoothing process was performed, and cylindrical flow extensions were incorporated to ensure fully developed flow inside the computational domain. The surface’s internal volume was discretized using FoamyHexMesh, part of OpenFOAM [25], and meshes of 1–1.3 million elements were generated with dominant-hexahedral core cells, low orthogonality and predominant alignment to the vessel surface. Rigid wall CFD simulations were solved by the OpenFOAM toolbox, using a finite volume method for incompressible flows set with second order spatial and time integration schemes. Blood was modeled as patient-specific, non-Newtonian fluid using the Bird-Carreau rheological model, with patient-specific hematocrit and total serum proteins and the assumed constant density of 1.05 g/cm^3^. To define boundary conditions, patient-specific flow waveforms obtained from US examinations were applied at the inlet of both the PA and DA (see mean BFVs in Supplementary Table 2), whereas a traction-free condition was implemented at the venous outflow. To avoid the initial transient effects, simulations were carried out for three complete cardiac cycles and only the third cycle was utilized for subsequent post-processing.

The post-processing of CFD results was performed using Paraview [26]. The velocity phenotype was qualitatively assessed by visualizing the velocity 3D streamlines, whereas the OSI was computed to evaluate flow disturbance, quantifying the percentage of the area that exhibited an OSI higher than 0.1. The calculation was conducted by taking into consideration a region of interest that spanned approximately 4 cm, including anastomosis, PA, V, and a small segment of DA. The analysis of morphological changes in AVF models generated at different time points involved extracting vascular lumen cross-sectional areas (CSAs) at 0.1 mm intervals along the venous centerline, starting from the center of the anastomosis and extending toward the vein.

### Statistical Analysis

The relationship between AVF sounds, hemodynamics and morphological changes was assessed qualitatively.

Moreover, high OSI (OSI > 0.1) area percentages were compared with the HLPR values obtained from the corresponding sound recordings. Data were divided into groups based on the extension of high OSI areas, setting the threshold at the overall median value. Boxplots were then generated to display the HLPR distribution within the two subgroups, and an independent t-test was also performed.

The relationship between US-based BFVs and acoustic outcomes was evaluated with a linear mixed-effects model to fit the data, highlighting the 95% confidence interval derived from bootstrap resampling. The model accounted for random patient effects, addressing the non-independence of multiple observations per patient. Specifically, the relationship between brachial BFV and maximum AVF sound peak amplitude was assessed at individual timepoints, as were changes in BFVs and changes in HLPR observed in two consecutive follow-ups.

The correlation, linear regression and the agreement between the HLPRs from sounds recorded by an expert and an inexperienced operators were assessed (results are available in Supplementary Fig. 1). Specifically, Pearson’s correlation coefficient (R) and the 95% confidence interval were computed, after checking for data normality using the Shapiro–Wilk test.

In all tests, statistical significance was set at *p* < 0.05 and data were considered independent from each other. All analyses were performed using R software, version 4.2.0.

## Results

### Computational Fluid Dynamics-Derived Hemodynamics and AVF Sounds

**Fig. 4:**
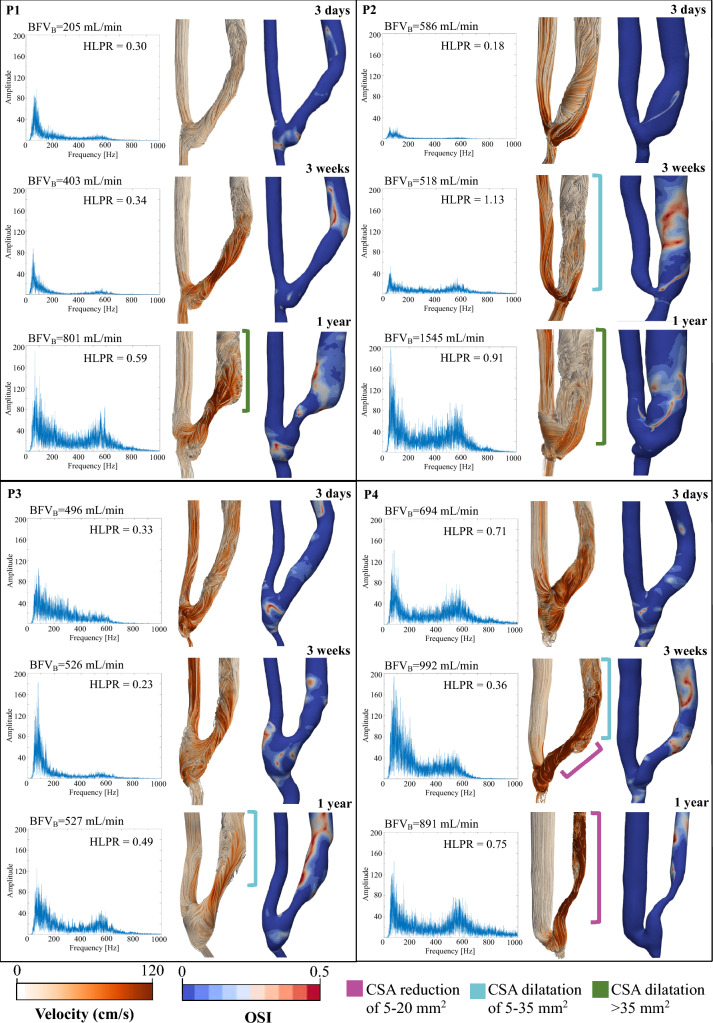
Representation of the evolution of morphology, velocity streamlines, OSI distribution and AVF sound frequency spectrum for each patient over time. *BFV*_*B*_ brachial blood flow volume, *CSA* cross-sectional area, *HLPR* high-low peak ratio, *OSI* oscillatory shear index, *P1* patient 1, *P2* patient 2, *P3* patient 3, *P4* patient 4

**Fig. 5:**
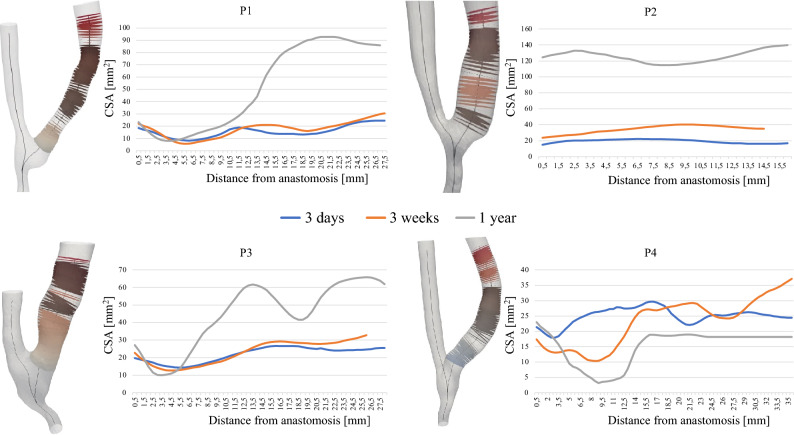
Representation of the evolution of AVF morphology in individual patients over time. *CSA* cross-sectional area, *P1* patient 1, *P2* patient 2, *P3* patient 3, *P4* patient 4

AVF sounds and respective longitudinal CFD-derived hemodynamic conditions of patients P1-P4, shown in Fig. 4, indicate that higher values of HLPR (> 0.35) are in general associated with complex blood flow velocity streamlines characterized by recirculation at the systolic peak, and corresponding extended regions of OSI > 0.1. On the contrary, in cases where the AVF sound is characterized by a predominance of low frequency peaks and values of HLPR lower than 0.35, the flow pattern of velocity streamlines is laminar and the OSI values are near zero. Moreover, the onset of high frequencies in sound (HLPR > 0.35), the complex blood flow features and the extended regions of high OSI are present in those AVFs which showed extensive venous remodeling (> 5 mm^2^ per CSA) between consecutive visits, whether due to significant dilatation or stenosis development. For the sake of completeness, we reported CSAs evolution during time for patients P1-P4 in Fig. 5.

The evolution over time of HLPR values and changes in the percentage of high OSI area are quantified and documented in Table 1. Notably, as HLPR increases due to the increase in high frequency components in sound from one follow-up to the next, also high OSI area expanded within the AVF; on the contrary, as HLPR decreases, the high OSI area decreases.

**Table 1:** Evolution of high-low peak ratio and areas of high oscillatory shear index in individual patients over time

	P1		P2		P3		P4	
	HLPR	% area OSI > 0.1	HLPR	% area OSI > 0.1	HLPR	% area OSI > 0.1	HLPR	% area OSI > 0.1
3 days	0.3	11.51	0.18	0.71	0.33	17.1	0.71	9.43
3 weeks	0.34	15.98	1.13	37.64	0.23	17.49	0.36	35.57
1 year	0.59	31.79	0.91	19.17	0.49	34.09	0.75	27.27

*HLPR* high-low peak ratio, *OSI* oscillatory shear index, *P1* patient 1, *P2* patient 2, *P3* patient 3, *P4* patient 4

**Fig. 6:**
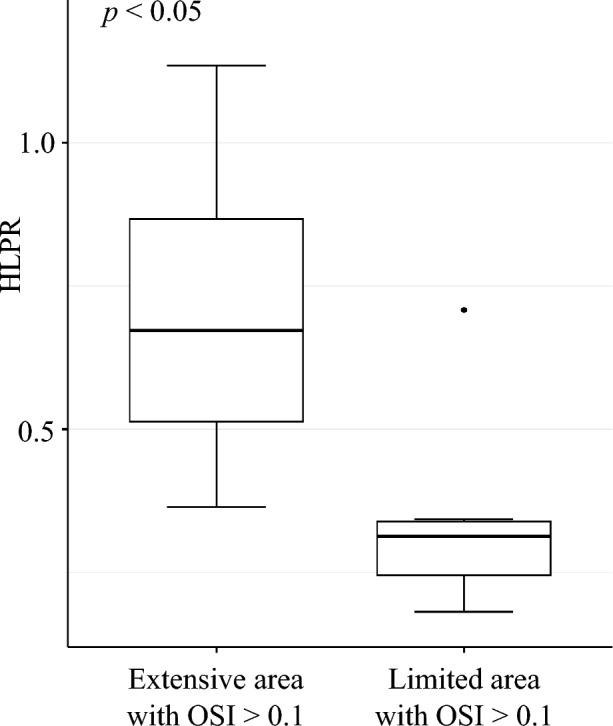
Distribution of HLPR within subgroups with extensive or reduced areas with OSI > 0.1. *HLPR* high-low peak ratio, *OSI* oscillatory shear index

Furthermore, significant differences were found in the median HLPR values of subgroups characterized by extensive or limited areas with OSI > 0.1, as shown in Fig. 6. Specifically, a median value of 0.67 [0.51 – 0.87] was found for the former group as compared to 0.31 [0.25–0.34] for the latter (*p* < 0.05).

### Ultrasound-Measured Blood Flow Volumes and AVF Sounds

**Fig. 7:**
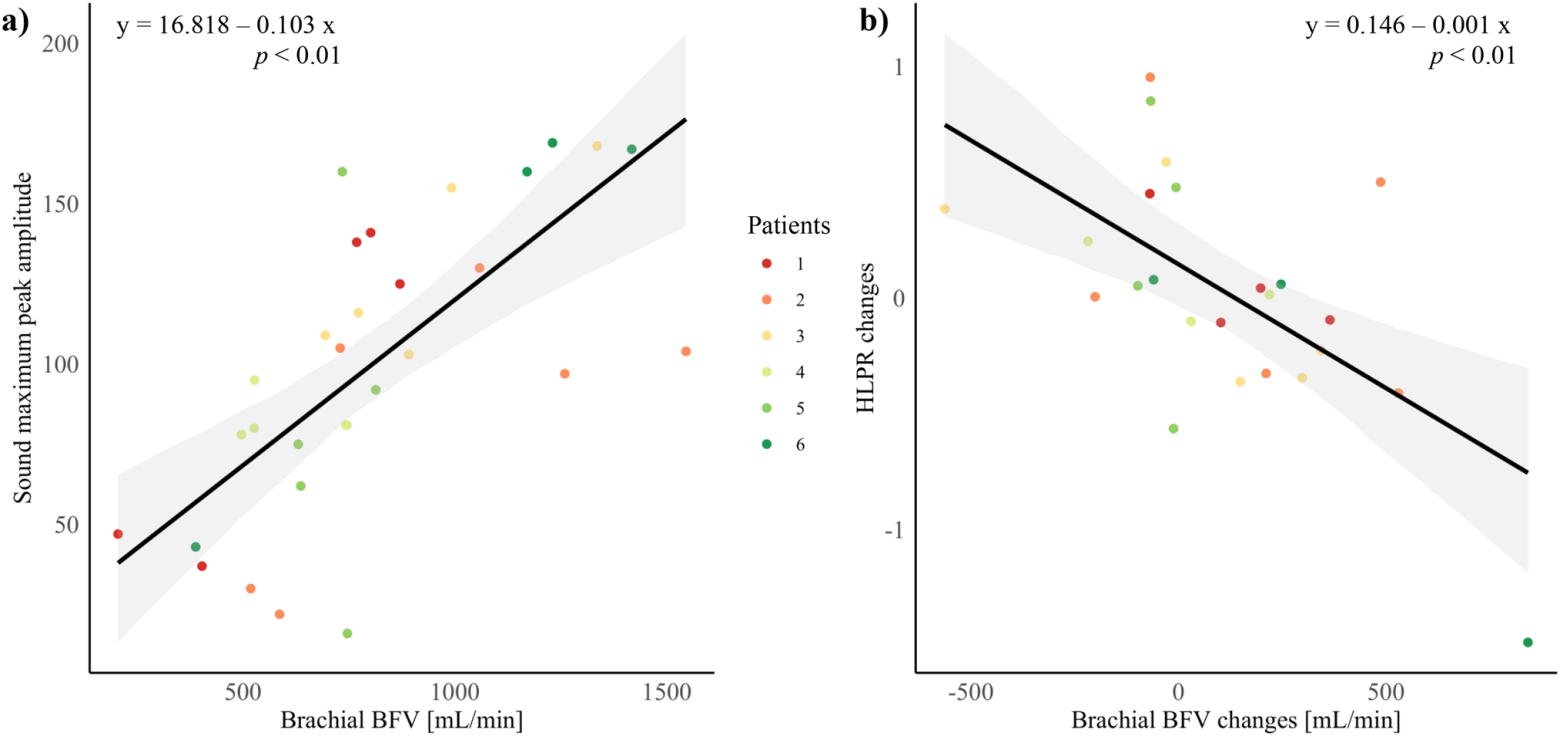
Relationship between US-based BFV and acoustic features in AVFs, assessed by a linear mixed-effects model. The black line represents the fitted values from the mixed-effects model, which includes random effects to account for multiple observations per patient. Each data point represents an individual measurement, color-coded by patient. The gray shaded area indicates the 95% confidence interval derived from bootstrap resampling. **a** Relationship between brachial BFV and maximum peak amplitude in sound. **b** Relationship between longitudinal changes in brachial BFV and in HLPR. *BFV* blood flow volume, *HLPR* high-low peak ratio

The relationship between US-based BFVs and acoustic features of AVF sounds is shown in Fig. 7. The direct proportionality between measured blood flow and the intensity of AVF sounds is supported by a statistically significant positive association (slope = 0.103, *p* < 0.01) between brachial BFV and the amplitude of the maximum peak in sound (Fig. 7a). The model accounted for random effects at the patient level, showing variability in intercepts among patients (patient standard deviation = 14.97), as well as residual variability within patients (residual standard deviation = 31.05). The relationship between BFVs and AVF sounds was further confirmed by the significative negative relationship between longitudinal changes in brachial BFV and in HLPR (slope = − 0.001, *p* < 0.01) during consecutive follow-up sessions (Fig.7b). Random effects analysis indicated minimal patient variability in intercepts (almost null patient standard deviation), highlighting a tightly controlled response within patients. Supplementary Table 2 provides a comprehensive breakdown of the US-acquired values of all BFVs for each patient across all follow-up sessions.

## Discussion

This study has shown that AVF sound analysis provided fundamental information on AVF hemodynamic conditions. Specifically, the statistically significantly higher HLPR values observed in AVFs characterized by extensive areas with OSI > 0.1 and recirculation in blood velocity streamlines, compared to AVFs exhibiting regular flow patterns and limited area of high OSI, revealed that the sound is an indicator of the presence of complex flow features. Moreover, sound intensity and longitudinal changes in HLPR were found to have a statistically significant association respectively with BFV and its longitudinal change over time, revealing that sound amplitude and frequencies are indicators of adequate BFV for hemodialysis treatment.

This is the first investigation offering a comprehensive longitudinal analysis of the relationship between sounds and hemodynamic conditions, but the first studies in the field date back to the 60s, and later several research groups have striven to identify the source of sound. Back in 1963, Roach et al. [27] noted that post-stenotic dilatation in adult dog arteries occurred when stenoses caused distal turbulence, as indicated by the presence of thrill and bruit. In 1990, Wang [28] used specialized signal processing methods to assess the spectral content of isolated diastolic heart sounds. His research revealed a typical rise in high frequencies in patients with occlusive coronary arteries, where turbulent flow fluctuations were present. Additionally, in a small group of patients, the spectra derived from diastolic acoustic signals recorded by a chest microphone closely matched theoretical predictions. Two decades later, Seo and Mittal found that bruits are mostly related to the time-derivative of the integrated pressure force on the post-stenotic segment of ideal arterial wall models [29]. Subsequently they developed and applied a hemoacoustic approach to a model problem of aortic stenosis murmur. The simulation results were verified and validated by a comparison with known solutions and experimental measurements [30]. In 2020 Ozedn [31] numerically investigated the impact of stenosis shape on pressure fluctuations downstream of the stenosis and the sound produced by a narrowed, idealized blood vessel through the sonification of pressure fluctuations. These approaches have only recently been applied to patient-specific problems by David Steinman’s group. In 2021 they performed the sonification of CFD-derived turbulent-like velocity data in a case of narrowing of the cerebral vasculature, producing an audio signal that reproduced the subjective sonance of the patient’s pulsatile tinnitus [32]. In the context of cerebral aneurysm, they offered a plausible mechanistic rationale for the high frequency sounds detected within these aneurysms. They proposed that a narrow-band, vortex-shedding flow pattern might induce more pronounced wall vibrations compared to a broad-band, turbulent-like flow [33].

Taken together, these studies’ findings are in line with current results regarding the association between high frequency sounds and disruptions in hemodynamics. However, it is important to note that most of these studies have primarily focused on a single type of geometrical remodeling in single or a few models of other anatomical regions, such as aortic or coronary stenosis, or cerebral aneurysms. In contrast, our research comprehensively investigated both vessel narrowing and dilation. Indeed, the presence of high frequency sounds in AVFs and CFD-based altered hemodynamics were detected in both stenosis and post-stenotic dilatation or in the presence of excessive dilatation. Furthermore, to the best of our knowledge, our study is the first to establish a relationship between recorded AVF sounds and hemodynamic conditions in patient-specific AVF models from subjects followed over time.

Specifically, our analysis revealed that dilatation of the venous downstream segment, observed in patients P1 and P3 one year after surgery (more pronounced in P1), was accompanied by an increase in turbulent-like blood flow, resulting in larger areas with elevated OSI and an increased HLPR due to high frequency components in sounds. The association between vascular remodeling, complex hemodynamics, and the presence of high frequency peaks in acoustic spectra was also confirmed in P4. Indeed, the patient developed stenosis (CSA reduction of 5–20 mm^2^) in the first tract of the venous segment (2–13 mm from the anastomosis) and post-stenotic dilatation (CSA dilatation of 5–35 mm^2^) 3 weeks after surgery. The stenosis progressed to a constriction of the entire vein one year later, which was more prominent in the initial segment, as shown by the CSA evolution in Fig. 5. These morphological alterations were associated with disturbed flow and high frequency components in the acoustic signals. Conversely, P2 experienced significant dilatation of the vein (CSA dilatation of 5–35 mm^2^ at 3 weeks and > 35 mm^2^ at 1 year), with a brachial BFV that almost tripled and with a disturbed flow phenotype that mainly occurred at 3 weeks and 1 year after surgery, accompanied by a prevalence of high frequency features in sounds.

In all cases, pronounced high frequency components in sounds, leading to an increased HLPR corresponded with the presence of extended OSI. Interestingly this happened when the AVF underwent remodeling from the previous visit. In contrast, reduced OSI was related to lower frequency and minimal vascular remodeling. These results are in line with the findings of Wang et al. [19, 34], who showed the presence of high frequency components in sounds from stenotic AVFs, characterized by the narrowing of the vascular lumen.

Despite the relationship between OSI and HLPR being evident, the correlation between these two indices was not statistically significant. This can be explained by the fact that both indexes are in a certain way normalized. On the one hand, HLPR entails the normalization of high and low frequencies. On the other hand, the OSI percentage is computed across a wide AVF region of the same dimension as the electronic stethoscope’s capture area, indicating the extent of high OSI areas rather than their intensity.

The clinical relevance of this work is related to the possibility to extract fundamental hemodynamic information from AVF sound recording. Specifically, HLPR parameter provide the indication of the presence of disturbed hemodynamic conditions, which deserve close monitoring as they may result in AVF stenosis or excessive dilatation. Furthermore, the sound amplitude can be used as surrogate for the presence of adequate blood flow to perform hemodialysis treatment, and longitudinal decrease in HLPR is indicative of decreased BFV through the AVF, which may ultimately result in the ineffectiveness of hemodialysis treatment. Therefore, on the basis of sound analysis it will be possible to identify patients who deserve close monitoring and timely intervention to preserve AVF patency and adequate BFV for the treatment. Unlike the current standard like US and the Krivitski method, sound measurement is fast, operator-independent, and requires no specialized skills. Developing a self-monitoring device for patients could enable at-home surveillance and, with an integrated alarm system, notify both patients and clinicians when hospital monitoring is necessary. This approach could streamline workflow at dialysis centers, allowing clinicians to conduct standard exams only when necessary, enabling timely intervention to prevent AVF failure. However, further clinical studies are needed to identify acoustic metrics linked to fistula status and malfunction degrees, which would also help differentiate between various complications. Indeed, while both stenosis and excessive dilatation increase high-frequency sounds, they represent different pathological conditions requiring distinct interventions. Stenosis causes the closure of the vascular access and the consequent impossibility to perform hemodialysis, while excessive dilatation could cause cardiac overload. Thus, current findings and this pioneering approach, alongside the real-world applicability of the procedures adopted, paves the way for a novel non-invasive and accessible method for AVF surveillance. Future implementation of sound-based AVF evaluation could significantly enhance clinical surveillance of AVFs, reducing the workload for operators and improving the effectiveness of AVF monitoring.

The current study has a few limitations that warrant discussion. The main challenge is the sample size of the study, which hinders comprehensive statistical analysis. Nevertheless, the outcomes of CFD simulations are non-trivial due to the difficulty in obtaining patient-specific images of multiple AVFs. This limitation could be mitigated in future studies by expanding the dataset, thereby facilitating more robust statistical analyses. The second limit of the study is the omission of vessel wall compliance in our CFD simulations. This limitation could be overcome in forthcoming research by incorporating fluid-structure interaction simulations. However, McGah et al. [35] established that rigid wall hemodynamic simulations can provide blood flow predictions with a level of accuracy comparable to fluid-structure interaction simulations. Another limitation is the possible presence of background noise in few sound recordings. This could possibly have decreased the correlation between hemodynamics and sound characteristics, which might have been even stronger, therefore highlighting the need to pay special attention to sound recording.

In conclusion, we have shown that sound can be exploited to extract fundamental information on the hemodynamic conditions of the AVF, providing indication of the presence of complex hemodynamic and adequate BFV to perform hemodialysis treatment. Sound analysis has therefore the potential to improve clinical AVF surveillance, towards an ameliorated AVF clinical outcome and a better quality of life of hemodialysis patients.

## Supplementary Information

Below is the link to the electronic supplementary material.

Supplementary file1 (DOCX 499 KB)

## Data Availability

All data are available on Zenodo platform (www.zenodo.org), under specific request to the authors.
