# Supplementary material for: The Potential of Sound Analysis to Reveal Hemodynamic Conditions of Arteriovenous Fistulae for Hemodialysis
Source: Ann Biomed Eng. 2024 Nov 1;53(1):230–40. doi: 10.1007/s10439-024-03638-2 (PMC11782333; doi:10.1007/s10439-024-03638-2)
Supplement: Supplementary file 1 — Supplementary file1 (DOCX 499 KB) [file 10439_2024_3638_MOESM1_ESM.docx]

# **SUPPLEMENTARY MATERIAL**

Supplementary Table 1

Examinations performed by each patient during individual visits

Abbreviations: MRI, magnetic resonance imaging; US, ultrasound; P1, patient 1; P2, patient 2; P3, patient 3; P4, patient 4; P5, patient 5; P6, patient 6.

Supplementary Table 2

Mean BFV values acquired by US in individual patients over time

Abbreviations: BA, brachial artery; BFV, blood flow volume; DA, distal artery; PA, proximal artery; US, Ultrasound; V, vein; P1, patient 1; P2, patient 2; P3, patient 3; P4, patient 4; P5, patient 5; P6, patient 6.

**Supplementary Fig. 1** AVF sound recordings dependence on operator experience. a) correlation and b) agreement between HLPR derived from the recordings performed by an expert (1) and an inexperienced (2) operator. Abbreviations: HLPR, high-low peak ratio.
